# Extranodal Nasal Type NK/T‐Cell Lymphoma of the Transverse Colon With Gastrointestinal Perforation in a 15‐Year‐Old: Durable Remission With Modified SMILE Chemotherapy and HSCT

**DOI:** 10.1155/crh/5148970

**Published:** 2026-07-02

**Authors:** Devon D. Christoffel, Ingrid S. Tam, Kris Milbrandt, Yue Wu, Rupesh Chawla, Victor A. Lewis, Paul R. D’Alessandro

**Affiliations:** ^1^ Department of Pediatrics, University of Saskatchewan College of Medicine, Saskatoon, Saskatchewan, Canada, usask.ca; ^2^ Department of Pathology and Laboratory Medicine, University of Saskatchewan College of Medicine, Saskatoon, Saskatchewan, Canada, usask.ca; ^3^ Department of Surgery, University of Saskatchewan College of Medicine, Saskatoon, Saskatchewan, Canada, usask.ca; ^4^ Jim Pattison Children’s Hospital, Saskatoon, Saskatchewan, Canada; ^5^ Department of Pediatrics, Cumming School of Medicine, University of Calgary, Calgary, Alberta, Canada, ucalgary.ca; ^6^ Alberta Children’s Hospital Research Institute, Calgary, Alberta, Canada, albertahealthservices.ca

**Keywords:** adolescent/young adult (AYA), case report, Epstein–Barr virus (EBV), NK/T-cell lymphoma, pediatrics

## Abstract

NK/T‐cell lymphoma (NKTCL) is an aggressive lymphoma associated with Epstein–Barr virus (EBV), with higher incidence in Asian populations. Typically, patients present in their fifth decade with extranodal disease in the upper aerodigestive tract. Pediatric NKTCL is rare, with no established pediatric standard of care treatment. We describe the case of a healthy 15‐year‐old Filipino–Canadian male who presented with transverse colon perforation and was diagnosed with extranodal EBV + nasal type NKTCL of the gastrointestinal (GI) tract. After a complicated initial surgical course, we elected to treat him using six cycles of modified SMILE (dexamethasone, PEG‐asparaginase, ifosfamide, and etoposide) chemotherapy and consolidative hematopoietic stem cell transplant (HSCT). The post‐HSCT course involved cytomegalovirus reactivation (buccal lesions, viremia); adenovirus reactivation (positive nasal swab, viremia); zoster reactivation; acute and chronic oral/upper GI graft‐versus‐host disease (GVHD); chronic skin GVHD (vitiligo); and avascular necrosis. At the most recent follow‐up, surveillance imaging and investigations at 2 years post‐transplant showed durable CR1 and 100% donor chimerism. To our knowledge, this is the fourth reported case of primary GI NKTCL in a pediatric/adolescent patient; the only patient treated with an asparaginase‐containing regimen; and the only patient to undergo consolidative HSCT. Our patient’s outcome supports the utilization of an aggressive approach to treat GI NKTCL in young, fit patients similar to Stage III/VI nasal NKTCL. Our case also highlights unique treatment considerations for adolescent cancer patients, including delayed diagnoses and lack of standardized protocols.

## 1. Introduction

NK/T‐cell lymphoma (NKTCL) is an aggressive lymphoma associated with Epstein–Barr virus (EBV) [1, 2]. Patients usually present in their fifth decade, with a higher incidence in Asian populations [1, 2]. NKTCL typically involves the nose, nasopharynx, and upper aerodigestive tract, but may involve other sites [1, 3]. Pediatric/adolescent NKTCL is rare [4]. Approximately 420 cases have been identified in patients aged 2–18 years [5–14]. Pediatric patients have a propensity for Stage I/II disease of the skin or head/neck [4]. Only three pediatric/adolescent cases have reported gastrointestinal (GI) involvement [15–18]. Prognostic risk stratification tools (i.e., PINK‐E score) have been developed, but generalizability to nonadult populations is limited (1% of the validated patient cohort was aged 14–20 years) [19].

Chemotherapy is the standard treatment, with the addition of radiation for some patients [2, 20]. Asparaginase‐containing regimens have shown the most efficacy [3, 17]. SMILE chemotherapy (dexamethasone, high‐dose methotrexate, ifosfamide, asparaginase, and etoposide) given for two to six cycles until complete response (CR1) has become a widely adopted adult regimen [3, 21]. No pediatric standard of care exists [2, 4]. Chemotherapy and radiation are recommended for Stage I/II nasal NKTCL, with alternating sandwich regimens described in pediatric series [2, 3]. Survival rates plateau in patients with higher‐stage disease, and early relapses have been noted, suggesting that consolidative treatment is needed [2]. Allogeneic hematopoietic stem cell transplant (HSCT) has improved outcomes, but data is limited [2]. Treatment‐related morbidity and mortality remain barriers to adopting this approach, as many elderly adults are not candidates for highly toxic protocols or HSCT [2, 20, 22]. Some clinicians advise treating extranasal GI NKTCL in selected young, fit patients aggressively with an asparaginase‐containing chemotherapy regimen and upfront allogeneic HSCT in CR1 [2, 16]. Similar approaches are utilized for Stage III/VI nasal NKTCL [2, 16]. We describe the fourth reported pediatric/adolescent case of extranodal nasal‐type NKTCL of the GI tract and the only case to utilize this approach.

## 2. Case Presentation

**FIGURE 1:**
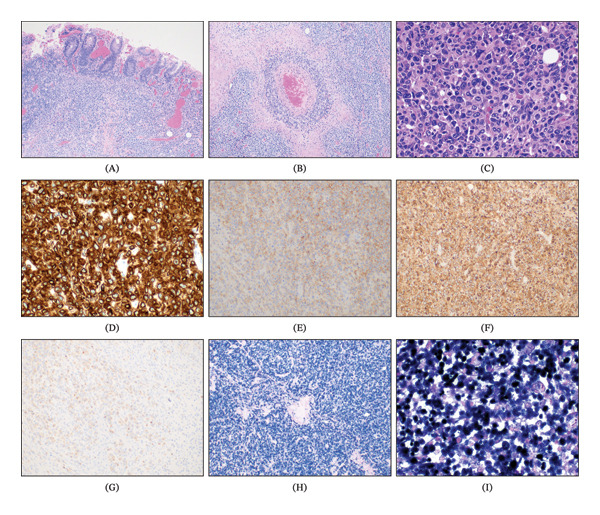
Morphology and immunophenotype of the extranodal NK/T‐cell lymphoma. (A) H&E (× 100) showing effacement of the mucosa with a destructive infiltrate. (B) H&E (× 100) highlighting angiocentric growth and necrosis. (C) H&E (× 400) showing atypical cells with irregular nuclear borders and abundant mitotic figures. (D–G) The atypical lymphoid cells were positive for CD3 (× 400), CD56 (× 200), Granzyme B (× 200), and TIA‐1 (× 200). (H–I) EBER in situ hybridization was diffusely and strongly positive in the neoplastic cells (× 100 and × 400).

The affected individual was a previously healthy 15‐year‐old Filipino–Canadian male who presented to the local hospital with six weeks of abdominal pain, hematochezia, and constitutional symptoms. Past medical/surgical/family/social history was noncontributory (specifically, no prior EBV infection or exposure, and no symptoms suggestive of recent sinusitis or sinopulmonary infection). Abdominal computed tomography (CT) showed transverse colon perforation with walled‐off collection. After transfer to a larger hospital, he underwent exploratory laparotomy, mesenteric mass biopsy, and colonic resection with end‐to‐end anastomosis. Pediatric oncology consultation and transfer to our tertiary pediatric centre were initiated on postoperative Day 8 when pathology showed extranodal EBV + nasal type NKTCL (Figure 1). At the time of transfer to our centre, he had developed an anastomotic leak and peritonitis, necessitating urgent laparotomy, takedown of the transverse colon anastomosis, and application of an open abdominal wound VAC dressing. Multiple laparotomies resulted in a diverting loop colostomy and mucous fistula. Staging CT and positron emission tomography (PET)/CT showed Deauville 5 colonic disease and Deauville 4 abdominal lymphadenopathy but no distant disease (Lugano IV). Cerebrospinal fluid and bone marrow studies showed no malignancy. EBV viral load was 20,570 IU/mL. The patient developed a postoperative intra‐abdominal collection (polymicrobial infection, cytology negative for malignancy). Institutional multidisciplinary tumor board recommended modified SMILE (mSMILE) chemotherapy with PEG‐asparaginase and consolidative allogeneic HSCT. Concerns existed regarding the potential toxicity of abdominal radiation given this patient’s surgical course.

mSMILE was initiated 37 days from diagnosis. Methotrexate was omitted due to the risk of accumulation into the intra‐abdominal collection. Treatment toxicities included transient peripheral neuropathy, attributed to metronidazole prescribed for intra‐abdominal abscess, and uncomplicated *Clostridium difficile* colitis. Biochemical CR1 (undetectable EBV viral load) was achieved after Cycle 4. Radiographic CR1 (Deauville ≤ 3) was favored after Cycle 2 (Deauville X due to persistent intra‐abdominal collection) and confirmed after Cycle 6. Methotrexate was reassessed prior to each cycle but was never introduced, as the intra‐abdominal collection did not resolve completely on cross‐sectional imaging until midway through Cycle 6. Repeat PET/CT, CT sinus/neck/chest/abdomen/pelvis, bone marrow aspirate/biopsy, and cerebrospinal fluid studies were negative for malignancy after six cycles.

He underwent allogeneic HSCT in CR1 18 days after count recovery from chemotherapy. Due to lack of donor availability and the goal for a consolidative transplant within the CR1 window, a haploidentical maternal donor was utilized. He was conditioned with busulfan, fludarabine, thiotepa, and rituximab and received post‐transplant cyclophosphamide, cyclosporine, and mycophenolate mofetil for graft‐versus‐host disease (GVHD) prophylaxis.

**FIGURE 2:**
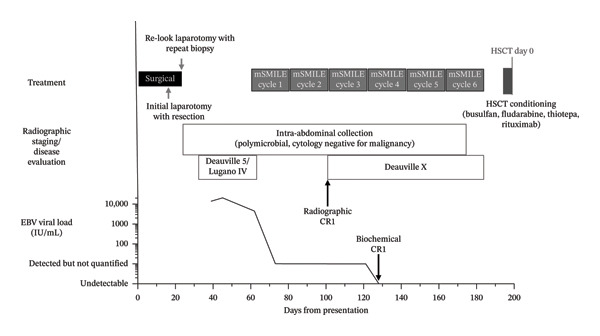
Diagnostic evaluations and treatment protocol, from time of presentation to haploidentical allogeneic hematopioetic stem cell transplant. EBV: Epstein–Barr virus; HSCT: hematopoietic stem cell transplant; mSMILE: modified SMILE chemotherapy, including dexamethasone, ifosfamide, PEG‐asparaginase, and etoposide. Note: original modification refers to substitution of PEG‐asparaginase instead of L‐asparaginase. Our team made an additional modification for our patient by omitting high‐dose methotrexate due to concern for potential accumulation into his intra‐abdominal collection.

The post‐HSCT course involved cytomegalovirus reactivation (buccal lesions and viremia); adenovirus reactivation (positive nasal swab and viremia); acute and chronic oral/upper GI GVHD; and chronic skin GVHD (vitiligo). Viral reactivations were treated with oral valganciclovir, intravenous ganciclovir, and intravenous cidofovir. Acute GVHD was treated with intravenous methylprednisolone, followed by taper of oral prednisone. Due to significant body image concerns on steroids, he was switched to oral ruxolitinib for chronic GVHD management. Additional complications within the first year post‐transplant included an adhesive small bowel obstruction, managed conservatively. Day +80 PET/CT showed no avid disease. Surveillance for disease recurrence has included serial EBV monitoring and cross‐sectional imaging, in addition to standard post‐HSCT care. Investigations 1‐year post‐transplant confirmed CR1 (undetectable EBV viral load; CT sinus/neck/chest/abdomen/pelvis negative for disease) with 100% donor chimerism. Eventually, his colostomy was reversed, and ruxolitinib was weaned. Within his second year post‐transplant, he was treated with intravenous zoledronic acid for avascular necrosis of his knees and intravenous acyclovir for cutaneous varicella zoster reactivation. At the most recent follow‐up 2 years post‐transplant, he remained disease‐free with excellent functional status and quality of life (Figure 2).

## 3. Discussion

We describe a rare case of NKTCL in an adolescent who presented with transverse colon perforation. NKTCL typically involves the nose, nasopharynx, and upper aerodigestive tract but may involve any site, including skin, salivary glands, testes, CNS, or GI tract [1, 3]. Rarely, disseminated cases present with hepatosplenomegaly, lymphadenopathy, and a leukemic phase [1]. Pediatric/adolescent NKTCL is rare, representing 0.2%–1.6% of newly diagnosed non‐Hodgkin lymphoma [4]. To our knowledge, only three pediatric/adolescent cases have reported GI involvement [15–18]. Ju et al. described a 13‐year‐old female with duodenal and jejunal disease who achieved clinical remission following COMP (prednisone, cyclophosphamide, vincristine, doxorubicin) chemotherapy. This patient did not receive any consolidative therapy and ultimately died from infection and a late relapse [16]. Yang et al. described a 15‐year‐old male treated with COTP (cyclophosphamide, vincristine, pirarubicin, and prednisone) who survived for 13 months [18]. Duan et al. described a 12‐year‐old male treated with surgical resection. The patient’s family declined any further treatment, and the patient died within 1 month [15]. All three cases utilized anthracycline‐based protocols and had unfavorable outcomes. In larger adult studies, anthracycline‐based regimens have been proven inferior compared to asparaginase‐based regimens [2]. Purportedly, inherent expression of p‐glycoprotein on NK cells facilitates anthracycline resistance [2].

Of note, we elected to modify the SMILE protocol via omission of high‐dose methotrexate due to the patient’s initial surgical course. As our patient had a persistent abdominal fluid collection, the use of high‐dose methotrexate (2 g/m^2^) presented a risk for further morbidity. Extravascular fluid collections are associated with delayed methotrexate excretion and subsequent renal toxicity in protocols using methotrexate >500 mg/m^2^ [23]. Even with this omission, we managed to achieve CR1. We took a similar approach regarding the decision to not use consolidative radiation therapy. In pediatric case series, some patients with early‐stage nasal‐type, extranodal NKTCL were treated using a “sandwich” chemotherapy/radiation therapy protocol that alternated two rounds of SMILE chemotherapy with two rounds of radiotherapy, prior to additional cycles of SMILE as needed in order to induce CR1. Some patients then underwent consolidative allogeneic or autologous stem cell transplant [4]. Given the proposed radiation field (i.e., whole abdomen due to upfront GI perforation and subsequent anastomotic leak) and the patient’s complex surgical history, our institutional multidisciplinary tumor board recommended against radiation, as risks of radiation‐related toxicities (including further GI perforation, sinusoidal obstruction syndrome, colitis, and bowel obstruction) outweighed the potential benefits. Additionally, there was concern that radiation‐related toxicity could delay allogeneic transplant (thus losing the CR1 window) or worsen morbidity post‐transplant.

Regarding the choice of consolidative transplant approach, there is a paucity of data regarding the use of autologous stem cell transplant and allogeneic HSCT for NKTCL. Given that many adult and elderly patients are poor candidates for highly toxic regimens and transplant, these approaches are neither widely used nor studied [2, 20, 22]. Autologous stem cell transplant has not improved outcomes and is not recommended upfront [2, 6]. Allogeneic HSCT has improved outcomes, but data is limited as it is associated with significant treatment‐related morbidity [2]. For this reason, allogeneic HSCT is often used in adults for relapsed or advanced disease. However, several authors advocate for the use of upfront consolidative HSCT in younger, fit patients with advanced disease (as presumably the benefit is preserved and the odds of toxicities are lower) [2, 16]. Our patient’s durable remission and manageable post‐transplant complications support this approach.

Lastly, our case highlights unique challenges faced by adolescent cancer patients, including delayed diagnoses; presentation to multiple loci of care; and lack of standardized treatment protocols. Our pediatric NKTCL data has been limited to case reports and series. Though large clinical trials exist, these have almost exclusively included adult NKTCL patients, many of whom were elderly. Understandably, it is difficult to extrapolate meaningful clinical standards from these trials to apply to younger patients [24].

## 4. Conclusion

We present the fourth reported case of pediatric/adolescent GI NKTCL. Our patient achieved CR1 following mSMILE chemotherapy and durable remission with consolidative allogeneic hematopoetic stem cell transplant. This favorable outcome adds to a growing body of knowledge regarding the utility of asparaginase‐containing regimens and upfront allogeneic hematopoietic stem cell transplant for patients with GI NKTCL, particularly pediatric/adolescent patients who are able to tolerate treatment regimens associated with greater toxicity.

## Author Contributions

Devon D. Christoffel, Ingrid S. Tam, and Paul R. D’Alessandro collected and analyzed the data and drafted the manuscript. Corresponding author, Paul R. D’Alessandro, had full access to all of the data in this study and takes complete responsibility for the integrity of the data and the accuracy of the data analysis.

## Funding

The authors have nothing to report.

## Disclosure

All authors reviewed the manuscript critically and provided final approval of the version to be published. All authors agree to be accountable for all aspects of the work. All authors have read and approved the final version of the manuscript.

## Ethics Statement

The patient’s parents provided informed consent, and the patient provided written assent for publication of this report as per institutional guidelines. A waiver was obtained from the University of Saskatchewan Research Ethics Board (REB #E‐Bio‐103.)

## Conflicts of Interest

Paul R. D’Alessandro has received an honorarium from Merck Inc. The other authors declare no conflicts of interest.

## Data Availability

The data that support the findings of this study are available from the corresponding author upon reasonable request.
